# Emerging Roles of RNA-Binding Proteins in Seed Development and Performance

**DOI:** 10.3390/ijms21186822

**Published:** 2020-09-17

**Authors:** Lijuan Lou, Ling Ding, Tao Wang, Yong Xiang

**Affiliations:** Shenzhen Branch, Guangdong Laboratory for Lingnan Modern Agriculture, Genome Analysis Laboratory of the Ministry of Agriculture, Agricultural Genomics Institute at Shenzhen, Chinese Academy of Agricultural Sciences, Shenzhen 518120, China; loulijuan@caas.cn (L.L.); dingling01@caas.cn (L.D.); wangtao01@caas.cn (T.W.)

**Keywords:** RNA binding protein, seed development, seed germination, seed dormancy

## Abstract

Seed development, dormancy, and germination are key physiological events that are not only important for seed generation, survival, and dispersal, but also contribute to agricultural production. RNA-binding proteins (RBPs) directly interact with target mRNAs and fine-tune mRNA metabolism by governing post-transcriptional regulation, including RNA processing, intron splicing, nuclear export, trafficking, stability/decay, and translational control. Recent studies have functionally characterized increasing numbers of diverse RBPs and shown that they participate in seed development and performance, providing significant insight into the role of RBP–mRNA interactions in seed processes. In this review, we discuss recent research progress on newly defined RBPs that have crucial roles in RNA metabolism and affect seed development, dormancy, and germination.

## 1. Introduction

RNA-binding proteins (RBPs) centrally regulate all aspects of RNA metabolism from synthesis to decay [1]. The functional characterization of RBPs and the resolution of their protein structures indicate that a variety of RNA-binding domains (RBDs) mediate direct interactions between RBPs and their target mRNAs. RBDs include the RNA recognition motif (RRM, also known as RBD or ribonucleoprotein (RNP) domain), the hnRNP K homology (KH) domain, the zinc finger motif, the pentatricopeptide repeat (PPR) motif, Asp-Glu-Ala-Asp (DEAD) boxes, Pumilio/FBF (PUF) domains, and the double-stranded RNA binding domain (dsRBD) [2,3,4,5]. Based on these RBDs, bioinformatic analyses have identified more than 800, about 250 putative, and 336 non-redundant RBP genes in the Arabidopsis, rice, and maize genomes, respectively. Many appear to be plant specific, highlighting their functional diversity and specificity [4,6,7]. Moreover, recent studies have used mRNA–protein interactome capture technology to discover many novel RBPs with non-canonical RNA-binding structures, providing unprecedented insight into the complexity of RNA metabolism in plants [8,9,10].

Transcriptome data have been used to identify more than 12,000 and 17,000 mRNAs in the mature dry seeds of Arabidopsis and rice, respectively [11,12]. These stored mRNAs have a pivotal role in the early stages of seed germination, as many proteins that function in germination are initially translated using stored mRNAs as templates. Germination would be halted if translation were inhibited. Thus, the fate of seed-stored mRNA is important for seed dormancy and germination [13]. Proteomic analysis of mature dry rice seeds has demonstrated that three different types of RBPs participate in seed germination: the KH domain-containing protein NOVA-like, putative RBP RZ-1A, and GR-RBP 1A. *GRP1A* and *RZ-1A* significantly increased during seed maturation and desiccation. Their encoded proteins potentially act as molecular chaperones to stabilize seed stored-mRNA and maintain its original integrity and translatability during seed dormancy and under various natural conditions [14,15]. Recent studies have also shown that several different RBPs, including PUF, dsRBD, CSP GRP, RZ, and PPR, have essential functions in seed dormancy and germination [16,17]. Our review therefore focuses on recent advances in understanding the emerging roles of RBPs in seed biology and emphasizes seed development, dormancy, and germination.

## 2. Structural Characteristics of RBPs

**Figure 1:**
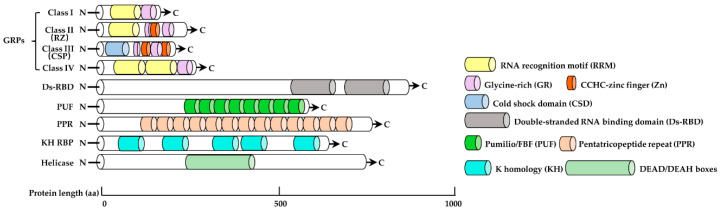
Structure diagram for different types of RNA-binding protein (RBP) from *Arabidopsis thaliana* described in this review (the **left**). The well-known RNA-binding domains (RBDs) and auxiliary motifs in plants (the **right**).

The RRM was the first conserved domain to be identified in RBPs and appears to be common to all eukaryotes [4]. In addition to the RRM, typical RBPs also harbor auxiliary domains rich in glycine, arginine, and serine. These are arranged in a variety of modes and determine the target specificity of individual RBPs [18]. The glycine-rich (GR) RNA-binding proteins (GRPs) are particularly well characterized and are divided into four subclasses (Figure 1). Class I and IV members contain one or two RRMs and a glycine-rich region at the N or C terminus, respectively. Class II members have one CCHC-type zinc finger inserted into the glycine-rich region of Class I; they are also named RZs because they are structural orthologues of RZ-1, a protein identified with a 60S RNP complex from tobacco nuclei [19]. Class III members are referred to as cold shock proteins (CSPs). They contain a cold shock domain (CSD) instead of an RRM, as well as two or more zinc fingers scattered within the glycine-rich region [20]. The CSD binds with RNA and with single- and double-stranded DNA in eukaryotes [21,22]. Protein structural investigations have revealed that, in addition to binding RNA, RRM can also mediate protein–protein interactions, through both of which heterogeneous RNP complexes are formed [23].

The dsRBD is the second most widespread RNA recognition motif and usually comprises 65 to 70 amino acids that form an αβββα fold [24]. How dsRBDs recognize their dsRNA cellular targets is not yet well understood (see review [25] for more details). PUF RBPs are highly conserved across eukaryotes and characterized by Puf domains (also called PUM-HDs; Pumilio-homology domains), whose name derives from the two founding members, Pumilio from *Drosophila melanogaster* and FBF (Fem-3 mRNA-binding factor) from *Caenorhabditis elegans* [26] (Figure 1). The canonical PUF RBP binds to a conserved UGURN_1-3_AU(A⁄U) motif in the 3′-untranslated region (UTR) of the target RNA, mediated by its eight sequential tandem PUF repeats. Each PUF repeat comprises approximately 36 amino acids and specifically recognizes one RNA nucleotide [27,28,29,30,31].

PPR proteins are composed of 2–30 repeats of approximately 35 amino acids each. They are encoded by the nuclear genome but predominantly localized to chloroplasts and/or mitochondria, where they regulate organelle-related RNA metabolism [32] (Figure 1). Based on variations in PPR unit length, PPR proteins are divided into two major types: the P class (P-motif: 35 amino acids long) and the PLS class (PLS-triplets: P-motif, with longer (L) and shorter (S) variants). With the exception of RNA editing, P-class proteins affect nearly all steps of post-transcriptional gene regulation. PLS-class proteins display C-to-U RNA editing ability, which mainly depends on the class-specific extended C-terminal domain. Also, the E or DWY domain in PLS-class proteins may contribute to their RNA association and to target specificity or editing [33].

The KH domain is another common structure in RBPs; it comprises approximately 70 amino acids and appears in multiple copies. KH domain-containing proteins primarily participate in the regulation of transcription and translation [34], and the KH domain itself has various roles, such as interacting with ssDNA or ssRNA and mediating protein–protein interactions [35]. DEAD box RNA helicases constitute a large family that contains 58 members and mainly functions in RNA metabolism and gene translation [36] (Figure 1).

## 3. PUF-Type RBPs in Seed Development and Performance

**Figure 2:**
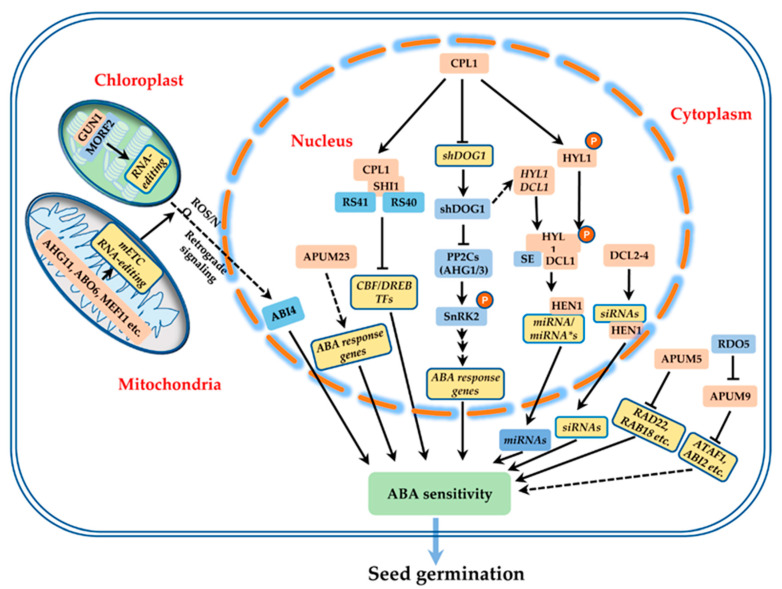
The model of various RBPs involved in ABA-related signaling pathway in the Arabidopsis seed germination and/or dormancy. All PRBs are showed in pink box, the targets of RBP are in yellow box, and the blue boxes are the other components in the signaling transduction. All proteins are normal font, and mRNAs are showed with italic font. Arrows with lines or dashed lines indicate the proteins and/or genes are directly or indirectly regulated by upstream factors, respectively. Lines with bars indicate the negative regulation in the signaling. P, phosphate; PP2C, protein phosphatase 2C; SnRK2, SNF1-related protein kinases 2; RD22, responsive to dehydration 22; RAB18, ABA-responsive gene 18; ATAF1, Arabidopsis transcription activation factor 1; ABI2, the phosphatase ABA Insensitive 2; ABI4, ABA-INSENTIVE 4 (a nuclear transcriptional factor); CBF/DREB, C-repeat binding factor/dehydration-responsive element (DRE) binding proteins.

PUF RBPs have been reported to affect RNA decay, translational repression, pre-ribosomal RNA processing, and chromosome stability [31]. Some Arabidopsis PUF RBPs (APUMs) are emerging as regulatory factors in seed development and performance (Table 1 and Figure 2). APUM5 was originally identified as causing reduced susceptibility to *cucumber mosaic virus* (CMV) in a T-DNA mutant screen [37]. *APUM5* was highly expressed in silique ends and was markedly upregulated after treatment with abscisic acid (ABA) and NaCl, suggesting that it may function in seeds in response to ABA and abiotic stress. Germination rate was hypersensitive to salt and osmotic stress in seeds that overexpressed *APUM5*, whereas *APUM5*-RNAi seeds showed increased tolerance of these stresses [38]. APUM5 was localized to the cytoplasm and shown to regulate abiotic stress response by binding to the 3′-UTRs of abiotic stress-responsive mRNAs and destabilizing them [39]. APUM23 and APUM24 have been reported to affect seed development. Together with APUM25, they are evolutionarily divergent and fail to group with other APUMs on the basis of their amino acid sequences and Puf domain locations [30]. They are localized to the nucleus and function in pre-ribosomal RNA processing. The *apum24* mutant is deficient in seed setting, whereas the *apum23* knock-out mutant develops normal embryos [16]. Their contrasting mutant phenotypes may arise from different RNA targets: an APUM24-containing complex interacted with INTERNAL TRANSCRIBED SPACER 2 (ITS2), whereas 18S rRNA was the target of APUM23 [40,41]. Additional pleiotropic defects were observed in the *apum23* null mutant and the *apum24* knock-down mutant, including significantly delayed germination and abnormal development of leaves and roots [40,42]. Notably, *apum23* null mutant seeds germinated more slowly than wild-type seeds, regardless of whether or not they were stratified, suggesting the involvement of APUM23 in seed dormancy release and germination [42]. Moreover, dysfunctions in *APUM23* also caused hypersensitivity to salt stress, with delayed germination and seedling establishment, possibly resulting from changes in the expression of genes involved in ABA biosynthesis and signaling [43].

Xiang et al. (2014) reported that members of another PUF subgroup, including *APUM9*, *APUM10* and *APUM11*, may function together with REDUCED DORMANCY5 (RDO5) in the regulation of seed germination and dormancy, as the expression of all three genes was significantly up-regulated in the *rdo5* mutant after imbibition. RNAi transgenic lines with simultaneous down-regulation of three APUMs exhibited enhanced dormancy compared with wild type. By contrast, the overexpression (OE) of *APUM9* resulted in reduced seed dormancy. Together, these results suggest that APUM9 has a strong negative effect on seed dormancy [17]. Interestingly, a transcriptome comparison of imbibed seeds identified six transcripts that were strikingly down-regulated in *APUM9*-OE plants compared with wild type. Five (*OZF2*, *ATAF1*, *FBS1*, *ABI2*, and *COL4*) were involved in the ABA signaling pathway. Moreover, APUM9 bound to the 3′-UTR of *FBS1* and *COL4*, subsequently destabilizing their neighboring *GFP* reporter gene in an *N. benthamiana* transient expression system. It therefore appears likely that APUM9 directly targets ABA signaling genes for degradation, thereby promoting the transition from dormancy to germination [44]. These APUMs function downstream of RDO5 and are required for the RDO5-mediated seed dormancy signaling pathway, as RNAi knockdown of *APUM9*, *APUM10*, and *APUM11* in an *rdo5-1* background completely rescued the reduced dormancy phenotype of *rdo5-1* [17]. In the future, it will be exciting to uncover other APUM9 targets and to decipher the mechanism by which APUM9 functions with RDO5 in dormancy regulation.

## 4. DsRBD-Type RBPs in Seed Development and Performance

DsRBD-type RBPs are mainly involved in post-transcriptional regulatory events such as the editing, trafficking, and portioning of substrate mRNAs and the biogenesis of various small RNAs. Based on the phylogenetic analyses of dsRBD sequence, 16 dsRBD-type RBP have been identified [45]. Six have been identified and characterized genetically, including RNA polymerase II C-terminal domain phosphatase-like protein 1 (CPL1), CPL2, HYPONASTIC LEAVES1 (HYL1), HUA ENHANCER1 (HEN1), Dicer-like protein (DCL1), DCL2 and DCL3. Abundant evidence confirms their involvement in seed development, germination, and dormancy regulation (Table 1 and Figure 2). In Arabidopsis, CPL1 and CPL2 are nuclear proteins that function as transcriptional repressors, which harbor the dsRBD at their C terminus and an RNA polymerase II C-terminal domain (CTD) phosphatase domain (CPD) at their N terminus [46]. The *cpl1* null and dsRBD defective mutants exhibit strong tolerance to ABA and NaCl stress during seed germination, indicating the requirement for CPL1, and especially the dsRBD, in typical seed germination behavior [47]. CPL1 interacts with the KH-domain protein SHINY1 (SHI1), which is also identified and named as HIGH OSMOTIC STRESS GENE EXPRESSION 5 (HOS5) or REGULATOR OF GENE EXPRESSION 3 (RCF3) through different screen strategies [48,49,50]. CPL1-HOS5(RCF3/SHI1) subsequently forms a regulatory complex by recruiting splicing factors serine/arginine-rich protein 40 (RS40) and RS41. This complex suppresses abiotic stress-responsive C-repeat binding factor (CBF)/dehydration-responsive element binding (DREB) transcription factors by inhibiting mRNA capping, thereby preventing the transition from transcription initiation to elongation [48]. By contrast to the *cpl1* mutant, the *cpl2* mutant with a defect in the dsRBD is hypersensitive to high salinity and shows a lower seed germination rate, suggesting that CPL1 and CPL2 have distinct functions in seed behavior under stress [51]. Interestingly, the KH domain-containing RBP PEPPER (PEP) was revealed to be another CPL1 interactor and shown to control ovule identity, the precursor of seeds [52]. Moreover, CPL1 has been shown to affect seed germination by positively regulating the nonsense-mediated decay (NMD) pathway. UPF1 is one of three key NMD factors, and the UPF1 null mutant displays elongated seeds, hypersensitivity of germination to ABA and glucose, and tolerance of germination to mannose, underscoring the significance of NMD in the control of seed germination [53]. CPL1 interacts with and dephosphorylates the DEAD box RNA helicase eIF4AIII, a core component of the exon junction complex (EJC) that recruits UPF1-3 to mRNA targets to trigger NMD [54]. These findings indicate that the different interactors of CPL1 and CPL2 may determine their target specificity during seed development and germination. They may act primarily through the recognition and elimination of mRNAs with premature translation-termination codons (PTCs) to prevent the production of truncated and potentially deleterious proteins [55].

Recently, CPL1 and CPL2 were reported to affect seed dormancy. The transcript of DELAY OF GERMINATION1 (*DOG1*), the major quantitative trait locus that specifically controls seed dormancy in *Arabidopsis*, is tightly controlled on different levels [56,57]. ETHYLENE RESPONSE FACTOR 12 (ERF12) and TOPLES (TPL) repress the expression of *DOG1* by directly binding its promoter region, while indirect regulation mode is adopted by histone methyltransferase KYP/SUVH4 [58,59]. On the other hand, CPL1 influences the alternative polyadenylation of *DOG1* mRNA. Alternative polyadenylation selectively occurs on the proximal transcription termination site (pTTS) and the distal TTS (dTTS) of the *DOG1* sense transcript, resulting in the production of *short DOG1* (*shDOG1*) and *long DOG1* (*lgDOG1*), respectively [60]. Indeed, seeds of the *cpl1-8* and *cpl1-9* mutants, defective in *CPL1*, exhibit priority polyadenylation of pTTS, increased expression of *shDOG1*, and enhanced seed dormancy [61]. However, the CPL1 cofactor that may determine *DOG1* target specificity during seed dormancy control has not been identified. More recently, the eceriferum (*cer*) mutant identified from a stem surface glossiness screen for wax-deficient mutants was shown to harbor a mutation in *CER11*, and *CER11* was demonstrated to be *CPL2*. Notably, the seed coat of *cer11*/*cpl2* showed reduced mucilage extrusion and deposition, as well as delayed columella secondary cell wall development [62]. Seed mucilage has been reported to affect germination, especially under conditions of limited water availability [63]. Moreover, the secondary cell wall acts as a barrier to block the exchange of gases and fluids between the mature seed and the environment, keeping the embryo dormant and preventing germination, and secondary cell wall defects reduce seed dormancy [64]. Distinct from its roles in the regulation of gene expression, CPL2 also functions in protein sorting in the *cer11* context, demonstrated by the fact that secretion of MUCILAGE-MODIFIED 2 (MUM2), a cell-wall modifying enzyme, is delayed in *cer11*/c*pl2* mutant seeds [62]. Therefore, it is likely that CRE11/CPL2 controls the maintenance of seed dormancy and the occurrence of seed germination through a previously unknown role in the cellular secretory trafficking pathway.

Most dsRBD-type RBPs, such as DCLs, HYL1/HYL1 homologs and HENs, are localized to the nucleus and influence the biogenesis of various small RNAs [65,66]. In addition to the dsRBD, some Arabidopsis DCLs contain DExH RNA helicase, RNase III, and/or Piwi/Argonaute/Zwille (PAZ) domains, which mainly function in small RNA processing. Arabidopsis HYL1/DRB1 and its four homologs DRB2–5 harbor two dsRBDs [45]. HYL1 is unusual, containing a nuclear localization sequence and six repeats of 28 amino acids with unknown function [67]. The HYL1 and DCL1 complex is exclusively involved in plant precise microRNA (miRNA) biogenesis. During this process, the first dsRBD of HYL1 interacts with a primary miRNA (pri-miRNA), and the second dsRBD mediates binding with SERRATE (SE) and DCL1 [68,69]. The DRB4–DCL4 complex primarily produces trans-acting (ta) siRNAs and viral siRNAs. DCL2 and DCL3 generate viral siRNAs independent of any dsRBP [70]. The *dcl1-11* mutant exhibits germination sensitivity to salt and osmotic stresses, and the germination of *dcl2*, *dcl3* and *dcl4* mutant seeds is sensitive to ABA [67,71]. HEN1 is a small RNA methyltransferase that mediates the methylation of miRNAs or siRNAs to protect them from degradation [72]. The *hen1-16* and hyponastic leaves1 (*hyl1*) mutants were identified from ABA sensitivity germination screens and are sensitive to salt and osmotic stresses during germination. HYL1 may affect the ABI5- or ABI3-mediated ABA signaling pathway to control seed germination and seedling establishment by repressing the activity of two mitogen activated protein kinases (MAPKs), SNP1 and MPK3, although the specific mechanism remains unclear [73]. Interestingly, the germination of *hyl1* and *dcl1-11* mutants was hyposensitive to glucose, which may be explained by alterations in 38 glucose-related pri-miRNAs and their corresponding miRNAs. Notably, the *hyl1* mutant showed lower expression of *ABI3*, *ABI4*, and *ABI5* after glucose treatment, contradictory to the *hyl1* ABA hypersensitive seed germination phenotype, suggesting an interaction between ABA, glucose, and miRNAs in the control of seed germination [74]. HYL1 activity is also regulated by phosphorylation/dephosphorylation: CPL1 and CPL2 promote its dephosphorylation and increase its activity, whereas SnRK2 and MPK3 may promote its phosphorylation [75,76,77]. The seed dormancy regulator DOG1 regulates miR156 and miR172 expression by promoting *DCL1* and *HYL1* expression in lettuce and Arabidopsis seeds [78]. Mutations in miRNA processing genes also affect embryo formation and development. The mutant of *DCL1* short integuments (*sin1*/*dcl1-7*), the hypomorphic mutant of *HEN1* (*hen1-8*), and the null mutant of *HYL1* (*hyl1-2*) are defective in asymmetric growth of the integuments, leading to abnormal ovule development and reduced female fertility [79,80]. Embryo patterning from the zygote to the globular stage is also severely affected in the *dcl1* and *hyl1-2* mutants. Transcriptome analysis has identified many miRNAs specifically expressed in the embryo, but their molecular mechanisms of action remain largely unknown [81].

In crops, rice miR156 and wheat miR9678 have been reported to function in seed dormancy through the regulation of the GA and GA/ABA pathways, respectively. Dysfunctions in one *MIR156* subfamily or overexpression of *MIR9678* increased seed dormancy and suppressed seed pre-harvest sprouting (PHS) [82,83]. These findings suggest that miRNA biogenesis mediated by dsRBD-type RBPs may have an important role in crop seed dormancy and germination.

## 5. GRPs in Seed Development and Performance

GRPs are involved in alternative splicing or transcriptional regulation. Many GRPs function in various stress-related seed germination processes (Table 1). The Arabidopsis genome encodes eight GRPs, and its class III includes four nuclear-localized members (CSP1–4) [84,85,86,87]. All *CSP*s are predominantly expressed in shoot apices and siliques at both the RNA and protein levels, but they have different roles in plant growth, seed development, and performance [88]. The overexpression of *CSP4* leads to reduced silique length and embryo lethality, whereas *CSP2* knock-down plants show changes in vegetative development and flowering control [84,87]. Overexpression of *CSP1* confers sensitivity to dehydration or salt stress with a delayed germination phenotype. By contrast, *CSP2*-OE lines exhibit accelerated seed germination under salt stress conditions [89]. CSP2 has also been designated as GRP2 [90]. Intriguingly, GRP2 only affects seed germination through an ABA-independent pathway. It also positively affects seed germination and seedling growth under cold stress [91]. Thus, although the spatial expression profiles of the *CSP*s are highly similar, their specific biological roles differ, and they deserve further study.

Another two of the Arabidopsis eight GRPs, GRP4 and GRP7, also affect plant stress response. *GRP4* is induced by cold stress, markedly repressed by salinity, and slightly repressed by dehydration. Intriguingly, *GRP4*-OE seeds exhibit delayed germination under salt and dehydration stress, but no noticeable differences were observed under cold or freezing stress [92]. In contrast to *GRP4*, *GRP7* is repressed by ABA, high salt, and mannitol treatment. Its knock-out mutant is hypersensitive to these stresses, displaying delayed seed germination and root development. Moreover, the *grp7* mutant has significantly higher expression of *RD29A* and *RAB18* than the wild type in response to stresses and ABA. It has therefore been proposed that GRP7 positively modulates gene expression in the ABA and stress signaling pathway [93]. However, this is inconsistent with the increased seed germination rate observed in another *GRP7* knock-out mutant under NaCl or mannitol treatment, which suggested that GRP7 had a negative effect on abiotic stress response. This discrepancy may have arisen from differences between the two mutants or from differences in the stress treatments or plant growth conditions; more accurate experiments are needed to assess the effects of GRP7 on stress tolerance [94]. In addition, GRP7 has been shown to promote seed germination and seedling growth under low temperatures [94].

Arabidopsis RZ-1A, RZ-1B, and RZ-1C are characterized by a zinc finger motif between the RRM domain and the C terminus. RZ-1B and RZ-1C fall into one phylogenetic clade, whereas RZ-1A belongs to a separate group, suggesting that they may have different biological functions [95,96]. *RZ-1A-*OE seeds showed delayed germination, whereas faster germination and earlier seedling onset were observed in the loss-of-function mutant after salt, dehydration, and freezing treatments. Furthermore, seed germination was affected by ABA or glucose, implying that RZ-1A negatively regulated seed germination through an ABA-dependent pathway [97,98]. By contrast, RZ-1B and RZ-1C redundantly function in the modulation of seed germination and other plant developmental processes under normal growth conditions. Their double mutant exhibited a significantly delayed germination rate and root and leaf defects compared to the wild type. This is consistent with the high levels of *RZ-1C* expression observed in embryos, endosperm of germinated seeds, and newly initiated leaves and root tips [96].

The rice genome encodes at least six GRPs, designated OsGRP1–OsGRP6. The overexpression of *OsGRP1*, *OsGRP4*, and *OsGRP6* in the Arabidopsis *grp7* knockout mutant rescued the cold-sensitive germination phenotype of the *grp7* mutant [99]. Both rice and wheat (*Triticum aestivum*) harbor three RZ proteins, referred to as OsRZ1–OsRZ3 and TaRZ1–TaRZ3, that are homologous to Arabidopsis RZs. The *OsRZ*s showed noticeable up-regulation only in response to cold stress, whereas the expression of the three *TaRZ*s was affected differently by high salt, drought, and cold stress. Only OsRZ2 affected seed germination under cold stress; the three *TaRZ* proteins had no significant effect under cold stress but had different effects on germination under high salt and dehydration [100,101]. These studies indicate that some GRPs and RZs of crop plants have important and diverse functions in abiotic stress response.

## 6. PPR-Type RBPs in Seed Development and Performance

PPR proteins are encoded by the nuclear genome but predominantly targeted to the chloroplasts and/or mitochondria, where they have a significant role in organelle RNA metabolism, biogenesis, and function [102]. Many PPR proteins are involved in embryo and/or kernel development, such as Arabidopsis GLUTAMINE-RICH PROTEIN23 (GRP23) and a series of embryo-defective (EMB) proteins; maize empty pericarp4 (EMP4), EMP5, and PPR8522; and rice opaque and growth retardation 1 (OGR1) [103,104,105,106,107,108]. Barkan and Small (2014) have presented a comprehensive review of the significance and possible biological mechanisms by which PPR proteins function in seed development [109]. Accumulating evidence indicates that PPRs are also involved in the regulation of seed germination (Table 1 and Figure 2). GENOMES UNCOUPLED 1 (GUN1) is a chloroplast-localized P-type PPR protein and an important regulator of plastid-to-nucleus retrograde signaling [110]. During chloroplast retrograde signaling, GUN1 directly interacts with the RNA editing factor MULTIPLE ORGANELLAR RNA EDITING FACTOR 2 (MORF2) to regulate plastid RNA editing and represses nuclear-encoded genes through ABI4 [111]. Seed germination of the *gun1* mutant is more sensitive to ABA treatment than *abi4*, suggesting that the germination regulation of *gun1* may be independent of ABI4. Furthermore, a later study showed that both ABA and sucrose delay the seed germination rate of the *gun1* mutant [112]. These results suggest a complex interplay between sucrose, ABA, and plastid signaling in seed germination. Many mitochondrially localized PPRs affect energy production and display an altered germination phenotype, especially in response to ABA. For instance, ABA hypersensitive germination 11 (*ahg11*), slow growth 1 (*slg1*), and slow growth 2 (*slo2*) show enhanced ABA hypersensitivity and delayed germination, and appear to act by editing mitochondrially encoded NAD genes of complex I (*nad1*, *nad3*, *nad4*, and/or *nad7*) [113,114,115,116]. Alternative splicing is another means by which some RRPs alter the expression level of mitochondrial electron transport chain (mETC) subunits. ABA overly sensitive 5 (ABO5) and PPR19 are responsible for the *cis*-splicing of *nad2* intron3 and stabilization of *nad1* exon2–exon3 transcripts, respectively [117,118]. The mitochondrial-localized DEAD box RNA helicase ABO6 also impairs the splicing of genes that encode complex I subunits (*nad1*, *nad2*, *nad4*, and *nad5*) [119]. Likewise, *abo5*, *ppr19*, and *abo6* mutant seeds also show delayed germination. Disruptions in PENTATRICOPEPTIDE REPEAT PROTEIN FOR GERMINATION ON *NaCl* (*PNG*) lead to an ABA hypersensitive phenotype with delayed germination, but it is unclear how PNG changes the transcript abundance of *nad1*, which encodes a complex I subunit [120]. Some PPRs regulate mETC activity by an unknown mechanism. For example, the *ppr40* mutant exhibits hypersensitivity of germination to ABA. No obvious alterations in RNA processes or in the abundance of mETC complex subunits were identified, but the *ppr40* mutant had a strongly reduced capacity for complex III electron transport and accumulated more reactive oxygen species (ROS) [121]. In contrast to the above-mentioned increased ABA sensitivity, PPR96 was demonstrated to negatively regulate the ABA pathway because the *ppr96* loss-of-function mutant exhibited an ABA insensitive phenotype with a faster germination rate. Future identification of the mitochondrial targets and molecular mechanisms of PPR96 in ABA tolerance will deepen our understanding of mitochondrial function, ABA, and seed germination [122].

Recently, the PPR protein MITOCHONDRIAL EDITING FACTOR11 (MEF11), also known as LOVASTATIN INSENSITIVE1 (LOI1), was isolated from a suppressor screen of the ABA biosynthesis-deficient mutant *aba3-1*. Dysfunction in *MEF11* led to increased seed dormancy. Remarkably, after one month of ripening and stratification, *mef11* mutant seeds still germinated slightly more slowly than the wild type. Moreover, hypersensitivity to the germination inhibitor paclobutrazol (a gibberellin biosynthesis inhibitor) and abiotic stresses (NaCl and mannitol) was also observed in *mef11* mutant seeds. These alterations in seed behavior may result from a loss of editing of the mitochondrial cytochrome c maturation FN2 (*ccmFN2*) gene, which encodes a cytochrome c-heme lyase subunit required for cytochrome c generation. Increased respiration and altered production of hydrogen peroxide (H_2_O_2_) and nitrogen monoxide (NO) were observed in *mef11* mutant seeds, as well as in the mitochondrial function-deficient *ppr* mutants described above [123,124].

Both ROS and NO function as positive signals that contribute to the transition from seed dormancy to germination, and they probably act through ABA signaling [125,126]. Additional findings suggest that PPR may affect ABA signaling through ROS and/or NO in mitochondrial retrograde signaling [127]. Recently, Nonogaki (2019) outlined a coherent molecular model that integrated mitochondrial function, ROS, NO, DOG1 and seed physiology [128]. However, more experiments are required to confirm and further develop this model. For example, with the exception of ABI4′s role as a retrograde regulator, little is known about the mechanisms of signal perception and transduction from the mitochondria to the nucleus in mitochondrial retrograde signaling.

In rice, the white stripe leaf (*wsl*) mutant exhibits chlorotic striations on leaves. The *WSL* gene encodes a chloroplast-localized PPR, and its mutant shows enhanced sensitivity to ABA, high salt, and sugar, with a delayed seed germination rate. Both mis-regulation of splicing in the chloroplast ribosomal protein subunit L2 (*RPL2*) transcript and decreased accumulation of plastid rRNAs and translation products were observed in the *wsl* mutant and may have caused its sensitivity to abiotic stresses [129]. OGR1 is targeted to the mitochondrion and acts by editing five mitochondrial transcripts (*ccmC*, *COX2*, *COX2*, *NAD2*, and *NAD4*). The *ogr1* mutant has pleotropic phenotypes, including delayed seed germination under normal conditions, but its mechanisms of action have not been described in detail [107]. In maize, chloroplast-localized PPR8522 and mitochondrion-targeted EMP4 have been reported to affect seed development and germination. Mutations in both genes are embryo lethal. Embryo rescue experiments showed that *ppr8522* and *emp4* embryos successfully germinated with sucrose supplementation, suggesting that a metabolic defect affects germination in the two mutant embryos [104,106].

**Table 1:** List of plant known RBPs involved in seed development and performance.

Type	Name	Species	Subcellular Localization ^a^	Seed Development	Seed Germination under Different Conditions					Seed Dormancy	Reference
					Normal	ABA	Salt	Osmotic	Cold		
PUF	APUM5	*A. thaliana*	Cytoplasm			√	√	√			[38,39]
	APUM23	*A. thaliana*	Nucleus		√	√	√				[42,43]
	APUM24	*A. thaliana*	Nucleus	√	√						[16,40]
	APUM9-11	*A. thaliana*	Cytoplasm/Nuclear/periphery		√					√	[17]
dsRBD	CPL1	*A. thaliana*	Nucleus	√	√	√	√			√	[46,47,52,61]
	CPL2	*A. thaliana*	Cytoplasm/Nucleus	√			√				[46,51,62]
	HYL1	*A. thaliana*	Nucleus	√		√	√	√			[67,71,73,80,81]
	DCL1	*A. thaliana*	Nucleus	√		√	√	√			[65,71,79,81]
	HEN1	*A. thaliana*	Nucleus	√		√	√	√			[66,71,80]
	DCL2-4	*A. thaliana*	Nucleus			√					[66,71]
GRP	CSP1	*A. thaliana*	Nucleus/Cytoplasm				√	√			[85,89]
	CSP2	*A. thaliana*	Nucleus/Cytoplasm				√		√		[84,89,91]
	CSP4	*A. thaliana*	Nucleus/Cytoplasm	√							[87]
	RZ-1A	*A. thaliana*	Nucleus/Cytoplasm			√	√	√	√		[95,97,98]
	AtRZ-1B	*A. thaliana*	Nucleus		√						[95,96]
	AtRZ-1C	*A. thaliana*	Nucleus		√						[95,96]
	GRP4	*A. thaliana*	Unknown				√	√			[92]
	GRP7	*A. thaliana*	Nucleus/Cytoplasm			√	√	√	√		[93,94]
	OsGRP1	*O. Sativa* L.	Unknown						√		[99]
	OsGRP4	*O. sativa* L.	Unknown						√		[99]
	OsGRP6	*O. sativa* L.	Unknown								[99]
	OsRZ2	*O. sativa* L.	Unknown						√		[100]
	TaRZ1	*T. aestivum* L.	Nucleus				√	√			[101]
	TaRZ2	*T. aestivum* L.	Nucleus/cytoplasm/ER				√	√			[101]
	TaRZ3	*T. aestivum* L.	Nucleus				√	√			[101]
PPR	EMBs	*A. thaliana*	Chloroplast	√							[103]
	GUN1	*A. thaliana*	Chloroplast			√					[110,112]
	AHG11	*A. thaliana*	Mitochondria			√	√	√			[113]
	SLG1	*A. thaliana*	Mitochondria	√		√	√	√			[114]
	SLO2	*A. thaliana*	Mitochondria			√	√	√			[116]
	ABO5	*A. thaliana*	Mitochondria			√					[118]
	ABO6	*A. thaliana*	Mitochondria			√	√	√			[119]
	PPR19	*A. thaliana*	Mitochondria		√						[117]
	PNG	*A. thaliana*	Mitochondria			√	√				[120]
	PPR40	*A. thaliana*	Mitochondria			√	√				[121]
	PPR96	*A. thaliana*	Mitochondria			√	√				[122]
	GRP23	*A. thaliana*	Nucleus	√							[108]
	MEF11	*A. thaliana*	Mitochondria		√	√				√	[123,124]
	WLS	*O. sativa* L.	Chloroplast			√	√				[129]
	OGR1	*O. sativa* L.	Mitochondria	√	√						[107]
	EMP4	*Z mays*	Mitochondria	√	√						[104]
	PPR8522	*Z. mays*	Chloroplast	√	√						[106]

^a^ Indicate the RBP subcellular localization verified with experiments.

## 7. Conclusions and Open Questions

Plant RBPs are a huge family with diverse biological roles. Nonetheless, only few of them have been functionally characterized with respect to seed biology, including seed development, dormancy, and germination regulation (Table 1 and Figure 2). Seed germination begins with the metabolism of stored mRNA in dry seeds after imbibition, and these early metabolic events may determine whether or not seeds germinate. RBPs appear to have an important effect on the fate of stored mRNA. Therefore, one pressing task is to systematically identify their substrate mRNAs and better understand their metabolism during early germination. As mentioned above, some RBPs display pleiotropic phenotypes, indicating that they may regulate different targets in a spatiotemporal model. RNA immunoprecipitation (RIP)-seq and/or cross-linking and immunoprecipitation (CLIP)-seq coupled with specific cell type isolation/fraction technology may help to elucidate their targets. In mammals, two newly established technologies, targets of RNA-binding protein identified by editing (TRIBE) and RNA tagging, provide alternative approaches for the genome-wide identification of plant RBP targets in vivo [130,131]. On the other hand, increasing evidence suggests that the regulation of RBPs is important, especially the control of their expression or protein activity during biological processes. Similar to human RNA-binding protein 25 (HsRBM25), Arabidopsis RBM25, a PWI and RRM domain-containing RBP responsible for alternative splicing of the ABA negative core component protein phosphatase 2C HABI, can be activated by ABA signaling not only at the transcriptional level, but also at the protein level through modulation of its phosphorylation status [132,133]. In maize, different types of RBPs, including RNA helicases, PUFs, and pre-mRNA-splicing factors, are preferentially modified by the cereal-specific small ubiquitin-like modifier (SUMO) variant DiSUMO-LIKE (DSUL) during embryogenesis. The DSULylation modification may affect activity and/or stability of the RBPs, resulting in translation/repression of specific mRNAs during embryo development [134]. Therefore, the characterization of RBP regulation at different levels is helpful for uncovering the biological mechanisms in which they are involved. We firmly believe that exciting progress on these questions is forthcoming and will integrate signaling pathways involved in mRNA metabolism during seed-relevant biological processes.
